# Canola produced under boreal climatic conditions in Newfoundland and Labrador have a unique lipid composition and expeller press extraction retained the composition for commercial use

**DOI:** 10.1016/j.jare.2020.05.002

**Published:** 2020-05-18

**Authors:** Albert Adu Sey, Thu Huong Pham, Vanessa Kavanagh, Sukhpreet Kaur, Mumtaz Cheema, Lakshman Galagedara, Raymond Thomas

**Affiliations:** a School of Science and the Environment, Memorial University of Newfoundland, Grenfell Campus, Corner Brook, NL, A2H 5G4, Canada; b Department of Fisheries and Land Resources, Government of Newfoundland and Labrador, Pasadena, NL, A0L 1K0, Canada

**Keywords:** Canola, Omega-3 fatty acids, Monoacetyldiacylglycerol, Non-solvent extraction, Lipid

## Abstract

The average fatty acid (FA) composition of canola oil is made up of 62% oleic acid (C18:1n9), 19% linoleic acid (C18:2n6), 9% linolenic acid (C18:3n3) and 7% saturated FA (SFA). We investigated whether boreal climate (7.5-17.2 °C) favorably altered the FA composition of canola. Results indicate that canola cultivated in boreal climatic conditions had approximately twice the levels of omega-3 FA (17-20%) compared to canola from other growing areas (9%). The presence of monoacetyldiacylglycerol (MAcDG), a unique class of triglyceride, is reported for the first time in canola cultivated in a boreal climate, and has the potential to reduce the risk of obesity and other health related diseases. We further demonstrated that a non-solvent based extraction method retained the novel lipid composition without reducing the quality of oil being produced. Our results contribute significantly to the understanding of lipid accumulation in the world's second most important oil crop when cultivated in a boreal or northern climate.

## Graphical abstract

**Figure:**
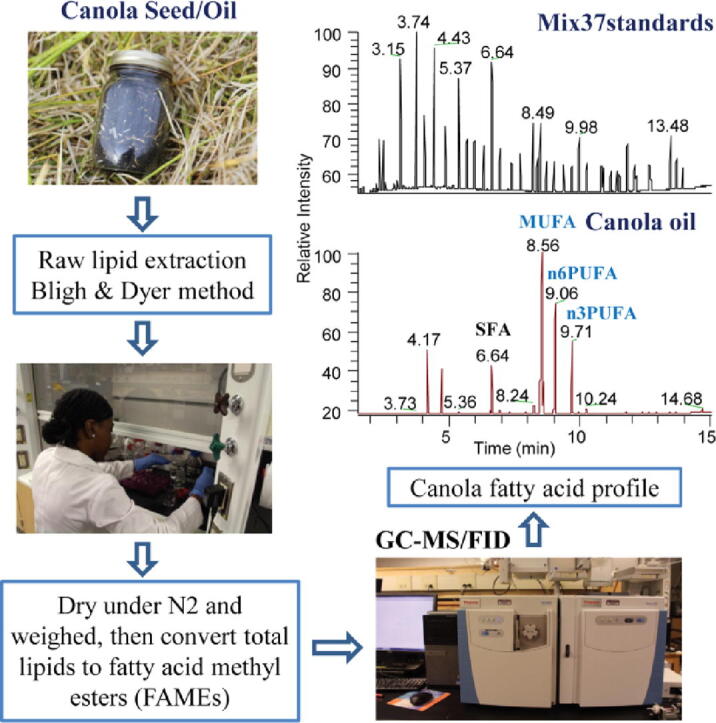

## Introduction

Canola (*Brassica napus* L.) is a member of the crucifer or rapeseed family developed in Canada by Keith Downey and Baldur Steffanson in 1975 [1], [2] by traditional plant breeding techniques as a low glucosinolate (e.g., 3-butenyl glucosinolate, 4-pentenyl glucosinolate, 2-hydroxy-3 butenyl glucosinolate, and 2-hydroxy-4-pentenyl glucosinolate) and euric acid (2%) rapeseed crop variety [1], [3]. The term ‘rapeseed’ refer to both high and low glucosinolate/erucic acid varieties used for edible and industrial applications [5]. Canola is known to contain about 45% oil, and the meal remaining after oil extraction contains about 40% protein [5], [6]. In fact, canola is the highest value oilseed crop grown in Canada contributing about $26.7 billion dollars to the Canadian economy [3] and is used in dietary supplementation in the form of salad dressing, baking, stir-fries, and margarines [7]. It is also one of the most widely used sources of biofuel [8]. The popularity and extensive use of canola in the food sector has resulted in the canola crop and industry rapidly expanding over the past 40 years, rising from the sixth largest oilseed crop to the second largest [9], [10] in the world.

Approximately 93% of the fats in canola are unsaturated fatty acids (FAs) [3] which have been reported to reduce blood cholesterol levels [2], [3], [11]. The polyunsaturated FAs (PUFAs) content in canola are made up of omega-3 (n3PUFA) and omega-6 (n6PUFA). Both omega-3 and omega-6 FAs are known as essential FAs that must be obtained from dietary sources such as canola, and are important in maintaining cardiovascular health, brain development, as well as modulating the immune response in human [3], [12]. Canola seed oil consist mainly of triglycerides which is an ester composed of one molecule of glycerol and three molecules of FAs esterified at each stereospecific numbered carbon (*sn*1, *sn*2 and *sn*3) positions respectively of the glycerol moiety. Based on the composition (carbon number) of the FAs, triglycerides can be further classified as short chain, medium chain and long chain triglycerides [13]. Recently, a unique form of triglyceride reported as monoacetyldiacylglycerol (MAcDG) has been gaining interest in the scientific community due to its potential applications in suppressing tumor growth, and treating inflammation-based illnesses such as sepsis, rheumatoid arthritis and asthma [12], [13]. As such, several recent patents have been granted for the use of MAcDG as the active ingredients in functional food formulations. MAcDG has so far been observed in various species including cold tolerant insects [14], deer antlers and moose meat [13].The structure of MAcDG is characterized by acetate at the *sn*3 position of the glycerol moiety. The presence of acetate at *sn*3 of glycerol give MAcDG unique properties and function; among them cold tolerance or cryoprotection during low temperature stress [14]. In addition, canola oil contains high amounts of bioactive compounds, such as polyphenols, phytosterols, tocopherols and other antioxidants [7], [15] and is also a rich source of vitamin E. Among these, phytosterols are plant steroids that are important to human health as their structure and function is similar to that of cholesterol, which is an integral compound in human and animal cell membranes [15]. This gives phytosterols the ability to reduce serum cholesterol, as well as low density lipoprotein (LDL) levels in humans; known risk factors for developing cardiovascular disease (CVD) [16]. While phytosterols are common in plants, they typically exist at low concentrations [16].

Canola is commonly cultivated under a temperature range between 12 and 30 °C [4], [17], [18]. Most of the global production of canola is concentrated in areas with dry weather (450-500 mm of rainfall per year) and short growing seasons (88-125 days) [4], [3]. In fact, canola cultivation in Canada has predominantly been conducted in the western Provinces due to the climatic conditions being more ideal for canola production compared to the climatic conditions in the North Atlantic region of the country. Newfoundland and Labrador (NL), for example has a boreal or northern climate with the average growth temperature during the growing season being 16 °C; which is on the lower end of the temperature spectrum (12-30 °C) suitable for canola production. Lower temperature can be one of the factors to alter the lipid composition of canola oil, as well as the oil content [19].

It is known that cool climatic conditions can shift the FA composition towards a greater increase in PUFAs; while higher temperatures favor the production of more saturated FAs (SFAs) [20], [21]. As such, it has been reported that under low temperatures the desaturation of oleic acid to linoleic acid, and that of linoleic to linolenic acid would be favored; resulting in altered levels of oleic, linoleic and linolenic acids in canola varieties produced under low growing temperature conditions [22], [23].

Canola was introduced for commercial production for the first time in 2016 in the Province of NL to address some of the challenges with food security in the Province. These challenges include an inadequate supply or production of food crops to feed the aging population as the Province relies heavily on importation. We hypothesize that canola produced under boreal climatic conditions in NL will have a unique lipid profile that could confer enhanced nutritional benefits as a high value niche crop. Furthermore, the processing method used for the extraction of oil may affect the lipid composition and FA content, influencing the quality of canola oil being produced [7]. The industrial processing of canola oil seeds involves pre-treatment (crushing/flaking and cooking), mechanical pressing and n-hexane extraction to recover the residual oil. As such, the FA composition of canola oil can be modulated by the processing methods used to produce higher value end products [7]. Consequently, the extraction yield and efficiency are important aspects of the bioprocessing method chosen, since they have a significant influence on the product quality and revenue. Two major processing methods used for producing canola oil are expeller press extraction (EE) and solvent extraction (SE). Commonly, the expeller method pre-heats the canola seeds between 135 °C and 160 °C before passing through a series of screw presses which crush the seeds in a rotating screw shaft. The use of high temperature in common expeller methods can affect the oil quality, although it is very efficient in extracting the oil from canola seeds. Understanding the impact/effect of climate in relation to the quality traits of rapeseed under different extraction conditions is necessary for improving the oil quality of NL canola. In view of this, the purpose of the research is to: (i) investigate the lipid profile in terms of FA composition, triacylglycerols and phytosterols of canola produced under boreal climatic conditions in NL and (ii) determine the effects of SE and EE processing on retaining the FA composition of the canola oil produced in NL.

## Materials and methods

### Study area

**Fig. 1:**
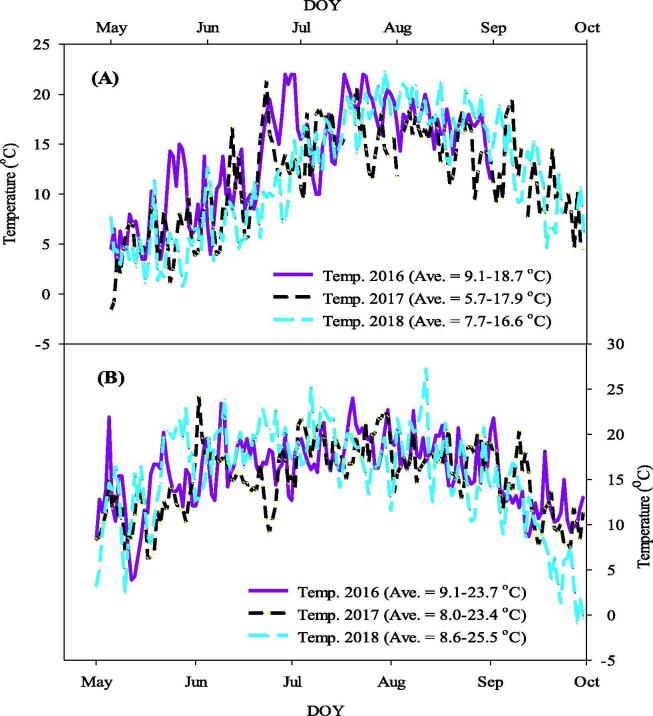
Variation in temperature across the three seasons used for canola cultivation reported in this study. Effect of growing season temperature on the lipid composition of canola grown under boreal climatic conditions in Newfoundland and Labrador (NL). The x-axis represents day of year (DOY) and y-axis represent temperature (°C). (A): temperatures under which NL canola was cultivated for the 3 years, (B): temperature of major canola growing areas in Manitoba (MB). Data used were the daily means adapted from Environment Canada for the respective canola growing areas.

Canola seeds were obtained from three (3) years canola trials following cultivation in podzolic soils at Pasadena (49°00′37.7″N 57°34′11.3″W) in NL for the years 2016-2018 under boreal climate condition. The field size was 28.3 ha and was seeded at a rate of 7.85 kg/ha using a Great Plains 1206 NT no-till drill (Great Plains Manufacturing, Salina, KS, USA). The row spacing was 191 mm and the same cultivation technique was used for all three growing seasons. The average growing temperature was 7.5-17.2 °C and rainfall 412.6 mm for the duration of the experiment (Fig. 1). Seeds used for analysis had a moisture content between 5.5 and 8.5% wet basis and were sieved to remove any debris collected at harvesting. Samples were transported to the lab in a hermetic zip lock bag, and the extracted oils kept in an air-tight glass jar.

## Oil extraction protocols

### Solvent extraction (SE) of canola oil from seeds

**Fig. 2:**
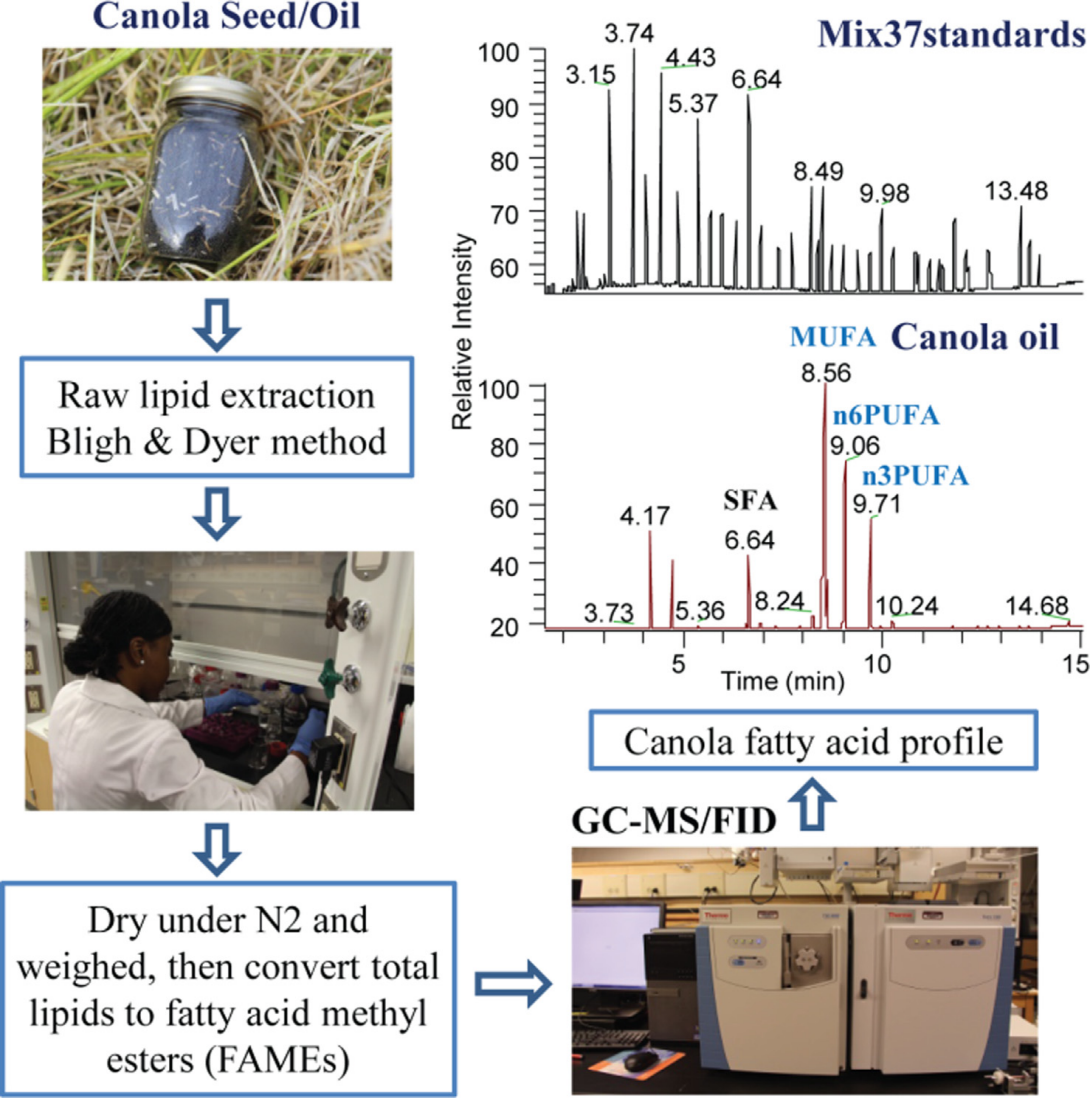
Solvent extraction (SE) of canola oil from seeds. Gas chromatography coupled with a flame ionization detector (GC-FID) and mass spectrometry (MS) was used to analyze the fatty acid (FA) composition. Results were obtained from the FA lipid profile. SE of lipids from canola seeds was done using the Bligh and Dyer method.

We sampled 300 g of seeds collected from transects across each replicate. The 300 g of seeds were homogenized to a fine powder in a cryomill (Reitch, Germany) and 300 mg of the homogenized seed powder was weighed and used for lipid extraction and analysis. Canola seed powder (300 mg) was mixed with 1.5 mL methanol (MeOH), 1.5 mL chloroform (CHCl_3_) and 1 mL water (H_2_O) according to the methods in [24] with some modifications. The sample mixture was thoroughly vortexed after each step of chemical addition, then centrifuged at 2500 rpm for 15 min in a Sorvall Legend XT/XF centrifuge (ThermoFisher Scientific, ON, Canada). The organic layer was then transferred to 2 mL vials, and dried under nitrogen (N_2_). Following drying, the samples were each reconstituted in 1 mL chloroform:methanol (1:1 v/v). An aliquot of the extraction (300 µL) was dried under nitrogen (N_2_), then converted to FA methyl esters (FAMEs) as follows: an internal standard (IS) consisting of 100 µL (C18 alkane at 0.5 mg/mL) was added together with 500 µL methanolic HCl (1.5 N), and the samples incubated in a pre-heated oven at 60 °C for 30 min. After incubation, 0.8 mL of distilled H_2_O was added to the cooled samples and the FAMEs extracted two times using n-hexane (500 µL each time). The samples were dried under nitrogen, then reconstituted into 100 µL hexane, transferred to inserts located in GC vials, and the FAMEs analyzed using a gas chromatography-mass spectrometry/flame ionization detector (GC-MS/FID). See Fig. 2 for depiction of SE extraction steps.

### Expeller press oil extraction (non-solvent extraction)

**Fig. 3:**
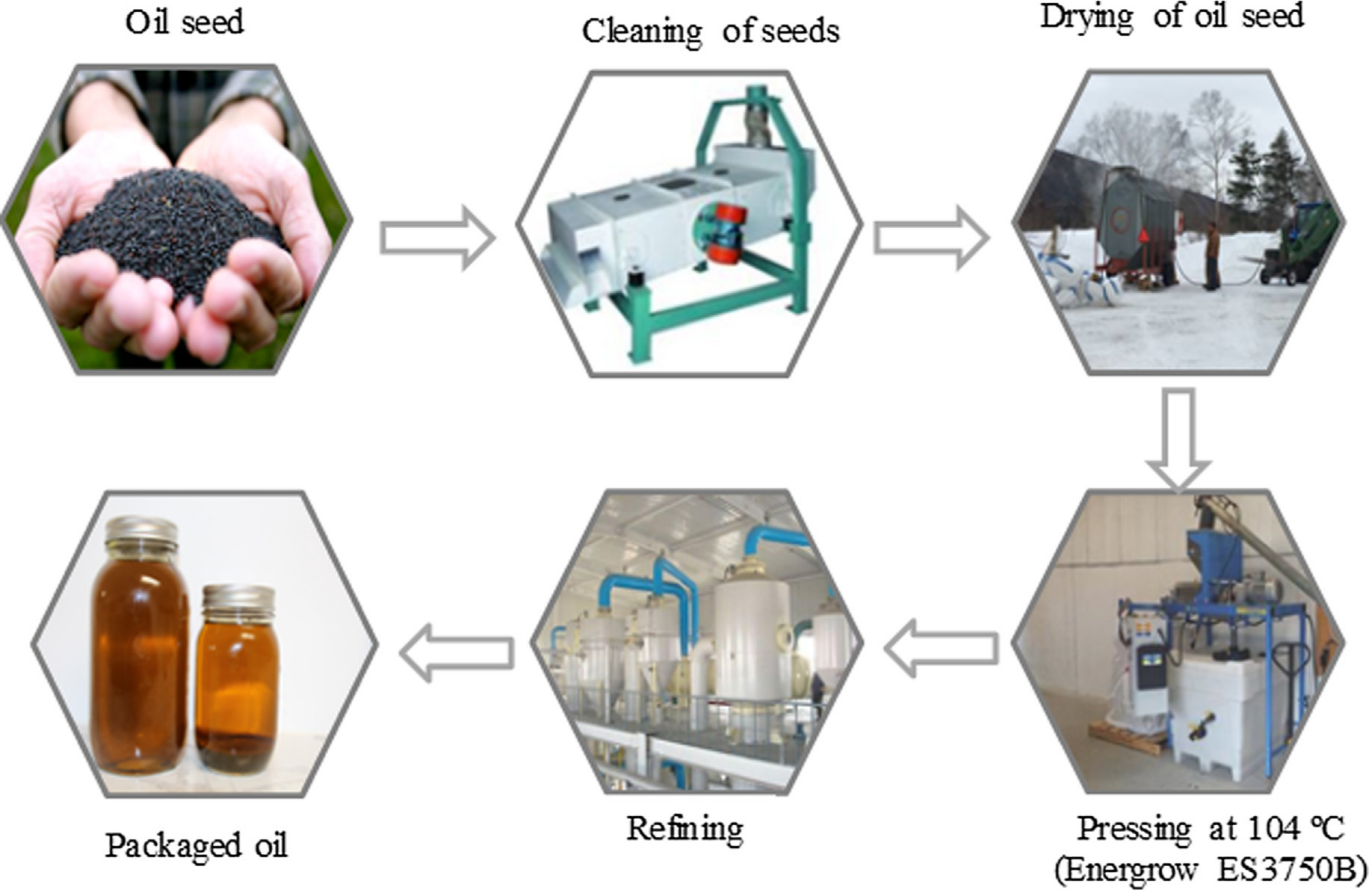
Expeller press oil extraction (non-solvent extraction). Extraction of NL canola oil using the Energrow ES3750B expeller press (104 °C). Detailed outline of extraction of oil from the canola seeds from harvesting to the finished product. Seeds were dried to moisture content between 5.5 and 8.5% wet basis (wb). Arrows indicates the direction of flow of the oil extraction.

Canola oil was mechanically extracted from the seeds using an Energrow ES3750B expeller press (Enegrow Inc., ON, Canada) at 104 °C (Fig. 3). However, it is worth noting that the temperature generated during the extraction was as a result of the friction between the canola seeds and the rotating drums in the pressing chamber, as compared to other commercial or industrial oil extraction operations. In this study, roasting or pre-conditioning of the seeds was not performed and oil was directly extracted from the seeds using the Energrow ES3750B expeller (Energrow Inc. ON, Canada) for the first extraction denoted as single pressed (EE 1). The canola meal (by-product) remaining after the first oil extraction (EE 1) was then fed back into the Energrow ES3750B expeller to extract the oil for the second time, denoted as double pressed (EE 2). At this point, ruptured surface area was increased to enable more oil to be extracted from the seeds for the second time (EE 2) under the same temperature (104 °C) condition. No food grade n-hexane was used during this process, which has often been used for industrial oil extraction. The FA composition of the expeller pressed (Fig. 3) oil was determined and compared with that of the SE procedure (Fig. 2) as described above.

### GC-FID and GC-MS analysis of FAMEs

GC-FID analysis was conducted on a Thermo Scientific Trace 1300 gas chromatography (GC) (Mississauga, ON, Canada) coupled to a flame ionization detector (FID) (Thermo Fisher Scientific, Waltham, MA, USA). Methylated FAs were separated with a BPX70 high resolution column (10 m × 0.1 mm ID × 0.2 μm) (Canadian Life Science, ON, Canada) using helium as the carrier gas at a flow rate of 1 mL/min. One (1 μL) of each sample was applied to the injection system in split mode (15:1) using a Tri-plus auto-sampler (Thermo Scientific, Burlington, ON, Canada). The oven temperature was programed as follows: the initial oven temperature of 50 °C was held for 0.75 min, then programmed to increase at 40 °C/min to 155 °C, then increased at 6 °C/min to 210 °C, then increased at 15 °C/min to 250 °C, where it was held for 2 min, total time: 17 mins.

GC-MS analysis was conducted using Thermo Scientific Trace 1300 gas chromatography coupled to a Thermo Scientific TSQ 8000 Triple Quadrupole mass spectrometer (Thermo Fisher Scientific, Waltham, MA, USA). The operational condition is reported elsewhere [25] and GC-MS was used to validate the presence of the FAs identified using the GC-FID. FAs in the samples were determined from comparison of retention times and mass spectra obtained from commercial standards (Supelco 37 component mix, Supelco PUFA No. 3, and Supelco FAME mix, C8-C24; Sigma Aldrich, ON, Canada) and the NIST database (ThermoScientific, Burlington, ON, Canada). The amounts of individual FAs identified were calculated using standard curves prepared from the standard mixtures, and values presented as either μg/g oil or % nmol of lipids for each sample.

### Lipidomic analysis of complex lipids in canola oils

In brief, canola oil was obtained from the field production (see Supplementary Table 1) using EE 1 and EE 2, and in laboratory using SE. From these homogenized on-site and off-site productions of oil, samples of 10 mg of each replicate were collected from the three different extraction methods, i.e., SE, EE 1 and EE 2. Samples were diluted in methanol to the final concentration of 1 mg/mL then 5 µL of sample was injected and analyzed by an ultra-high performance liquid chromatography (UHPLC) using a ThermoScientific™ UltiMate™ 3000 System coupled to a Q-Exactive orbitrap mass spectrometer (Thermo Fisher Scientific, Waltham, MA, USA). A C30 reverse phase (C30RP) high resolution column was used for lipid separation by liquid chromatography. The mobile phase system consisted of solvent A (acetonitrile: water 60:40 v/v) and solvent B (isopropanol:acetonitrile:water 90:10:1 v/v/v) both containing 10 mM ammonium formate and 0.1% formic acid. The column was re-equilibrated to starting condition (70% solvent A) for 5 min prior to each new injection.

Samples were ionized via heated electrospray ionization (HESI) prior to analysis by high resolution accurate mass tandem mass spectrometry (HRAM-MS/MS). The analysis was done in the positive ion mode according to our previous published methods [13], [25]. The following parameters were used for the Q-Exactive orbitrap mass spectrometer - sheath gas: 35; auxiliary gas: 2; ion spray voltage: 3.2 kV; capillary temperature: 300 °C; S-lens RF: 50 V; mass range: 200-2000 *m*/*z*; full scan mode at a resolution of 70,000 *m*/*z*; top-20 data dependent MS/MS at a resolution of 35,000 *m*/*z* and collision energy of 35 (arbitrary unit); isolation window: 1 *m*/*z*; and automatic gain control target: 1e5. The instrument was externally calibrated to 1 ppm using ESI positive calibration solutions (ThermoScientific, MO, USA). Tune parameters were optimized using a mixture of lipid standards (Avanti Polar Lipids, Alabama, USA).

Identification of the individual phytosterols and glycerolipids, most abundantly triacylglycerols in oil content, was accomplished using Lipid Search version 4.1 (Mitsui Knowledge Industry, Tokyo, Japan) and manual confirmation by X-Calibur 4.0 (ThermoScientific, MO, USA) software packages. Comparisons of retention times and mass spectra using commercial standards (Avanti Polar Lipids, Alabama, USA) was used to assist with identification and quantitation according to the well-recognized rules established by tandem mass spectrometry [13], [25].

### Data analysis

Four replicates of the seeds and EE 1 and EE 2 oil samples collected each year over the three (3) years were used for analysis. One-way analysis of variance (ANOVA) was used to determine either the effects of growing seasons or processing methods (SE, EE 1 and EE 2) on the canola lipid composition. In cases where treatment effects were significant, the means were compared with Fisher’s Least Significant Difference (LSD), α = 0.05. Principal component analysis (PCA) was also carried out to show the segregation of the lipids into different quadrants based on treatments. Analysis was performed using XLSTAT (Premium Version, Addinsoft, Paris, France) and figures prepared with SigmaPlot 12.5 software programs (Systat Software Inc., San Jose, CA).

## Results and discussions

### Effect of boreal climate on the FA composition of NL grown canola

**Fig. 4:**
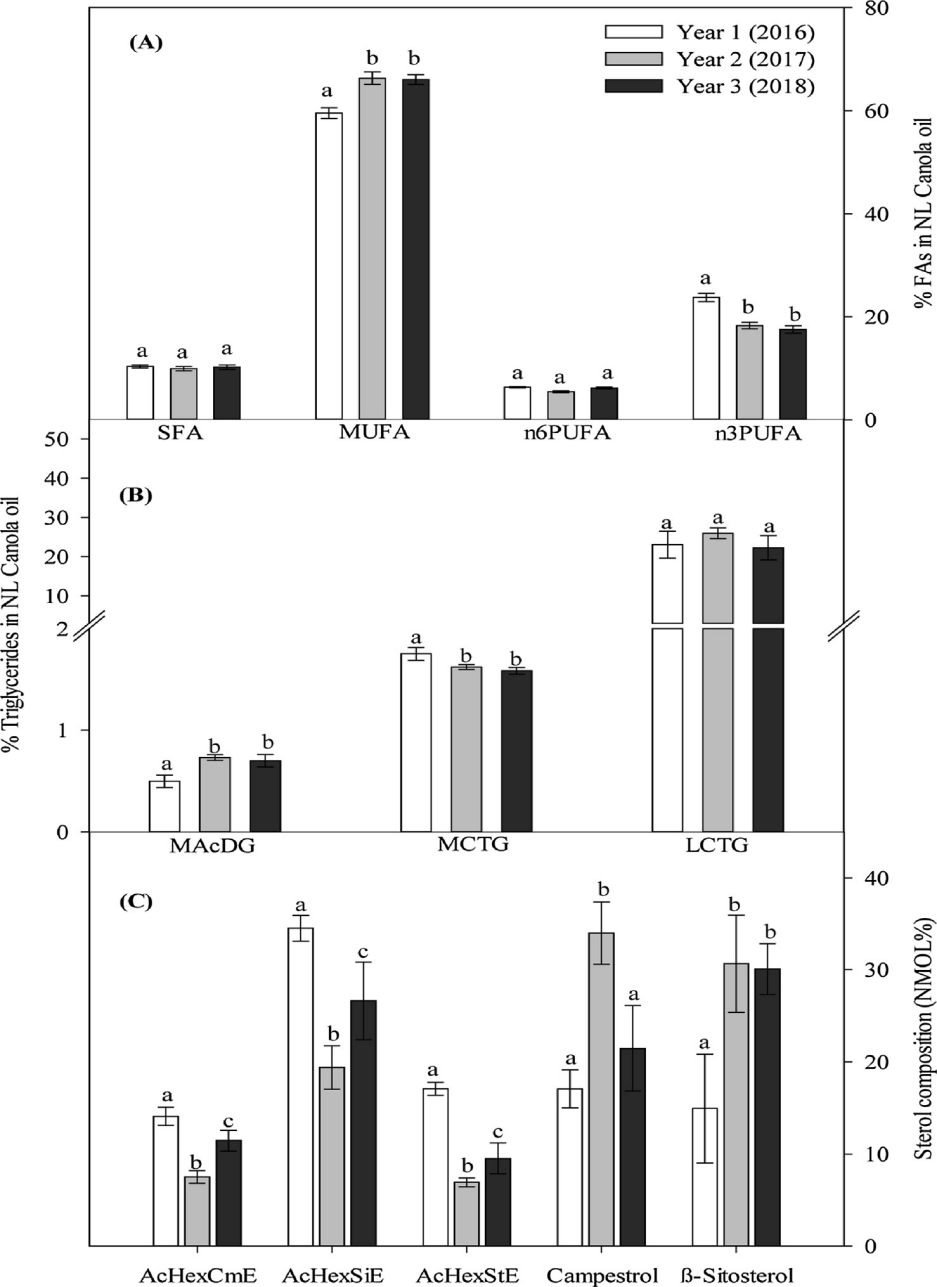
Effect of boreal climate on the fatty acid (FA) composition of Newfoundland and Labrador (NL) grown canola (A): percentage of FA (% FA) composition in NL canola oil (Saturated FA (SFA), monounsaturated FA (MUFA), omega-6 polyunsaturated FA (n6PUFA), omega-3 polyunsaturated FA (n3PUFA)), (B): represents the neutral lipids in the oil (monoacetyldiacylglycerol (MAcDG), medium chain triglycerides (MCTG), long chain triglycerides (LCTG), and (C): phytosterols composition: Acyl hexosyl campesterol (AcHexCme), Acyl hexosyl sitosterol ester (AcHexSiE), Acyl hexosyl stimasterol ester (AcHexStE), campesterol and β-Sitosterol).

The FA composition of canola is generally made up of 62% oleic acid (C18:1n9), 19% linoleic acid (C18:2n6), 9% linolenic acid (C18:3n3) and 7% SFAs (predominantly C16:0 and C18:0) [26]. However, cool climatic conditions can shift the FA composition of canola towards a greater increase in PUFAs; while higher temperatures tend to favor the production of more SFAs [20], [21]. In view of this, we observed that cultivation of canola in NL under boreal climatic conditions dramatically altered the FA composition. This alteration was consistent over the 3 growing seasons where an average growing temperature of 7.5-17.2 °C was recorded in NL (Fig. 1A), compared to the average growing temperature of 8.6-24.2 °C observed in most canola growing areas such as Manitoba (Fig. 1B) for the 3 growing seasons we evaluated. We observed that NL canola contains twice the level of n3PUFA (20%) in relation to the standard canola oil (9%) following cultivation under boreal climate conditions. NL canola also contained 64% of monounsaturated FA (MUFA), 6% of n6PUFA and 10% of SFA in the total FA composition (Fig. 4). Conversely, the standard canola composition consists of 7% SFA, 19% n6PUFA, 9% n3PUFA and 62% MUFA [3], [26].

**Fig. 5:**
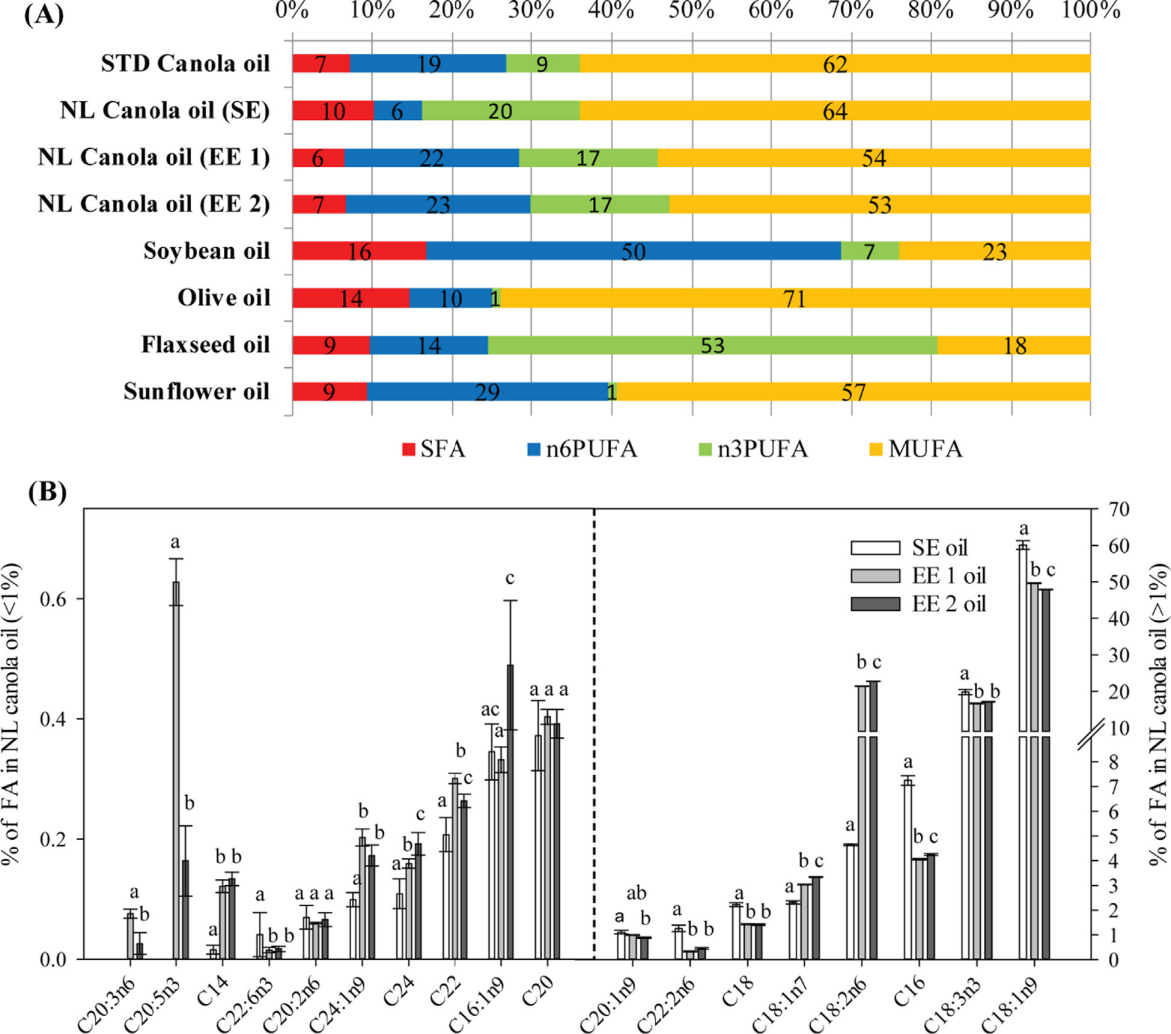
Effect of extraction methods on the fatty acid (FA) composition of canola produced in boreal climate. Comparison of the dietary fats of Newfoundland and Labrador (NL) canola oil in relation to standard canola oil and other common edible plant seed oil showing relationships with the level of omega-3 FA (A). The legend represents saturated FA (SFA) i.e., C14; C16; C18; C20; C22; C24, polyunsaturated FA in the form of omega-6 (n6PUFA) i.e., C18:2n6; C20:2n6; C22:2n6, polyunsaturated FA in the form of omega-3 (n3PUFA) i.e., C18:3n3; C20:6n3 and monounsaturated FA (MUFA) i.e., C24:1n9; C16:1n9; C20:1n9; C18:1n7; C18:1n9. (B): FA composition of NL canola oil after expeller press or solvent extraction. SE = solvent extracted, EE 1 = seed oil extracted once with expeller press (single press), EE 2 = seed oil extracted twice with expeller press (double pressed). Fig. (A) was adapted from: Canola Council of Canada and modified to include NL canola oil. The oil content presented in (A) is universally regarded or accepted to represent the approximate FA composition of the various oils presented for comparison purposes with NL canola. We used the universally accepted Bligh and Dyer and FA methyl ester (FAME) methods to analyze the FA composition consistent with the methods used to conduct reference profiles in the other oils presented for comparison.

It has been reported that under low temperatures, the desaturation of oleic acid to linoleic acid, and that of linoleic to linolenic acid would be favored; resulting in altered levels of oleic, linoleic and linolenic acids in canola varieties produced under low growth temperature conditions [22], [23]. This is consistent with the profile we observed for NL canola, where increase desaturation of linoleic acid to linolenic acid was observed to occur concomitant with cultivation in boreal climatic conditions (Fig. 3, Fig. 4). To the best of our knowledge, the percentage of omega-3 FAs in NL canola oil is only second to flaxseed oil in terms of content in plant seeds (Fig. 5A), and therefore surpassed the average omega-3 content in all canola oil previously reported globally. Climate is a major factor affecting the oil content and lipid composition of canola cultivated under cool climatic condition as suggested by Baux, Hebeisenb and Pellet [23], Commission [26] and Brown, Davis, Lauver and Wysocki [4]. As such, a decrease of 0.68% in canola oil content was observed for each 1.0 °C increase in post-anthesis temperature [17]. This suggest canola produced under boreal climatic conditions in NL has a unique omega-3 profile, and the potential to be a high value niche product for the traditional and functional foods market. Diets in western cultures tend to be low in omega-3 FAs, and as such low omega-3 intake has been reported to be a risk factor in poor fetal brain development, CVD, immune response and inflammation-based illnesses [27], [28]. Thus, the increased level of omega-3 FAs of canola grown in boreal climatic conditions could be uniquely helpful in improving heart and/or brain health and boosting the immune system; since omega-3 FAs are not synthesized by the body, and therefore needs to be obtained from dietary sources [13], [25], [27], [29]. Other studies suggest, daily consumption of omega-3 could also improve the amount of calcium in the body and reduce the risk of osteoporosis [30], [31].

The effect of the boreal climatic condition present in NL did not only alter the lipid composition of the canola oil but also increased the yield of canola seeds to 2.1 t/ha (Supplementary Table 1) compared to the average typical yield for Atlantic Canada (1.85 t/ha) [32]. This is consistent with reports in the literature that canola crops cultivated in cooler climates can produce 20-30% more yield, and higher oil content than crops produced in warmer temperatures [4]. For example, Si & Walton (2004) reported a reduction in total yield by 0.29 t/ha for every 1 °C increase in growing season temperature. Furthermore, Yaniv, Schafferman and Zur [21] reported a decrease in PUFA with increasing temperatures. This is congruent with our findings where we observed an increased in n3PUFA when cultivated at an average growing temperature of 7.5-17.2 °C compared to average canola growing temperatures of 12-30 °C across the major canola growing regions. Nuttall, Downey and Raney [19] found that temperature alone may not be the ideal abiotic factor that influence canola yield, but this could be coupled with other factors. At this point, we can only speculate that the podzolic soil conditions under which the canola was cultivated in NL might have also aided in the increased yield compared to production in solonetzic soil in British Columbia (BC), Alberta (AL), Saskatchewan (SK) and Ontario (ON) where large amounts of the world’s canola is cultivated [3].

### MAcDG as a functional lipid in NL canola oil

**Fig. 6:**
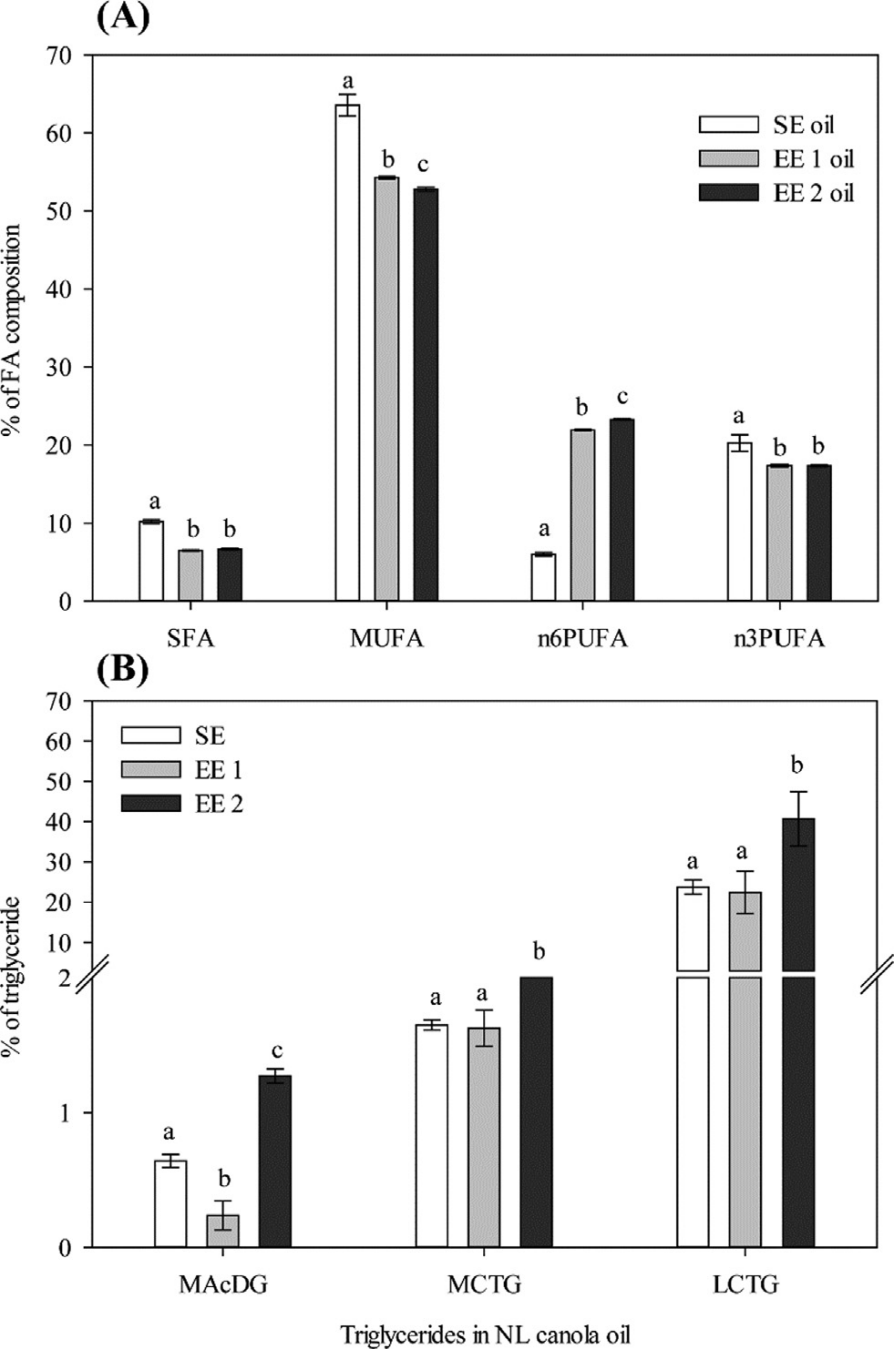
Effect of canola oil extraction on the fatty acid (FA) composition and monoacetyldiacylglycerol (MAcDG) content of canola produced in boreal climate of Newfoundland and Labrador (NL). (A): Effect of the different oil extraction methods has on the FA composition of NL canola oil. White bars represent solvent extracted (SE) oil, light gray represent first expeller extracted (EE 1) oil and dark gray bars represent second expeller extracted (EE 2) oil. Values were measured in percentage of fatty acid (% of FA) composition in NL canola oil. Saturated FA (SFA), monounsaturated FA (MUFA), omega-6 polyunsaturated FA (n6PUFA), omega-3 polyunsaturated FA (n3PUFA). Results were expressed as means ± standard error, n = 4 samples per treatment. Different lowercase letters above the bar indicate a significant difference (α = 0.05). (B): Effects of processing extraction methods on the level of MAcDG in canola oil. Relative quantification of triglycerides in canola oil based on their extraction methods for SE, EE 1 and EE 2 at temperature (104 °C), MAcDG, medium chain triglycerides (MCTG) and long chain triglycerides (LCTG). Values were measured in % of triglycerides in canola oil. Results were expressed as means ± standard error, n = 4 samples per treatment. Different lowercase letters above the bar indicate a significant difference (α = 0.05).

Here in, we report for the first time the presence of MAcDG in canola oil (Fig. 4B and Fig. 6B, Table 1). This observation further reiterates that the lipid composition of canola seed and oil following cultivation under boreal climatic conditions in NL is unique. MAcDG represent a unique subclass of triacylglycerols composed of acetate at the *sn*3 position of the glycerol moiety instead of other common medium (6-12 carbons) and long chain FAs (13-26 carbons) [13]. Eight molecular species of this unique subclass of triglycerides were observed in NL canola oil Table 1. MAcDG was observed in all three growing seasons and levels were consistent and represented approximately 1% of the total triglycerides present in the seeds and oil. The medium chain triglycerides (MCTG) level doubled that of the MAcDG with the long chain triglycerides (LCTG) representing the largest proportion of the total triglyceride content in the seeds (Fig. 4B).

**Table 1:** Quantity of MAcDG molecular species obtained using different extraction methods.

RT	Molecular species	*m*/*z* Prec ion [M + NH_4_]^+^	SE (µg/g of oil)	EE 1 (µg/g of oil)	EE 2 (µg/g of oil)
19.94	18:1/16:0/2:0	654.5667	5.51 ± 1.57	*nd*	14.1 ± 0.76
19.47	18:2/16:0/2:0	652.5510	2.90 ± 1.00	*nd*	14.0 ± 1.13
17.23	18:3/16:0/2:0	650.5354	*nd*	*nd*	9.57 ± 5.24
17.54	18:1/18:1/2:0	680.5823	271 ± 23.12	105 ± 34.14	401 ± 7.84
19.38	18:2/18:1/2:0	678.5667	205 ± 11.7	81.4 ± 28.85	360 ± 13.51
18.91	18:2/18:2/2:0	676.5510	130 ± 5.04	68.7 ± 27.14	357 ± 20.19
18.44	18:3/18:2/2:0	674.5354	33.6 ± 2.64	12.6 ± 9.15	115 ± 3.90
17.89	18:3/18:3/2:0	672.5197	*nd*	*nd*	18.8 ± 0.78

RT = retention time, Prec ion = precursor ion [M + NH_4_]^+^, SE = Solvent extraction, EE 1 = single press expeller extraction and EE 2 = double press expeller extraction, *nd* = not detected. Results were expressed as means ± standard error values reported as µg per g of canola oil.

Table 1 indicates the quantity of MAcDG in µg/g of NL canola oil. MAcDG was reported to occur in the metabolism of living organisms and animals at low temperature and in response to cold acclimation, freeze tolerance or cryoprotection [14], [33], [34]. The low temperature in the boreal ecosystem of NL may explain the presence of these compounds in canola acclimation to the cool climatic conditions (average growing temperatures of 7.5-17.2 °C). Interestingly, MAcDG has very high bioactivity at low concentrations, similar in range to that reported for signaling lipids [13]. Considering the improved lipid profile reported in this study including the discovery for the first time of MAcDG in canola, the possibility exist that the improved lipid profile of NL canola may present opportunities for its use as a raw material in the production or innovation of novel functional foods.

### Phytosterols in NL canola oil

Phytosterols, are natural plant-derived compounds that are structurally related to cholesterol and present in all fats and oils, providing the main source of sterols in a consumer’s diet. Among such commodities, canola and corn oils contain the highest amounts of phytosterols [35], [36]. Consistent with this information, we observed high levels of phytosterols in canola in this study. Most notably, we observed significant levels of acyl hexosyl campesterol ester (AcHexCmE), acyl hexosyl β-sitosterol ester (AcHexSiE), acyl hexosyl stigmasterol ester (AcHexStE), campesterol (CmE) and β-sitosterol esters (SiE) in NL canola oils. However, the levels varied significantly with growing temperatures across the three study years, and were the most variable of the lipids reported in this study in response to the average growing temperatures (Fig. 4C). Phytosterols have been reported to contribute to antioxidative function and thermal stability in vegetable oils [7]. Our results indicated that CmE, and SiE were the predominant types of phytosterol in canola produced in the second year of production when the average temperature was 11.6 °C, while AcHexSiE predominate the first year when the average temperature was 13.9 °C (Fig. 4C).

From a health perspective, phytosterol is known to reduce the risk of coronary heart disease, one of the leading causes of morbidity and mortality, in North America. Furthermore, SiE, has been reported to be effective in reducing the number of tumors in colon cancer [37], [38], [39], [40], as well as lowering plasma LDL levels; reducing the risk for developing CVD [25], [27], [28], [41], [42], [43]. The high levels of phytosterols present in NL canola and the fact that the growing temperature significantly modulates their levels suggest the potential exists for NL canola to be a unique source of dietary phytosterols with applications in functional foods or niche secondary product formulations or development. NL has the highest rate of morbidity and mortality associated with coronary heart diseases of all the Provinces in Canada [44]. As such, increased commercial production and consumption of NL canola oil and/or NL canola-based products could form part of a long-term strategy to improving personal and population health in the Province.

### Effect of canola oil extraction on the FA composition of canola oil

In this study, we evaluated whether SE, EE 1 and EE 2 methods were effective in retaining the novel lipid profile present in NL grown canola. We used EE 1 and EE 2, a non-solvent based commercial canola oil extraction technique compared to the renowned Bligh and Dyer method in assessing the effectiveness in retaining the novel lipid profile present in NL canola. Results from this study indicates the presence of 18 different types of FAs in the profile of NL canola regardless of the extraction method used (Fig. 5B). These FAs include: C14:0, C16:0, C18:0, C20:0, C22:0, C24:0, C16:1n9, C18:1n9, C18:1n7, C20:1n9, C20:3n6, C24:1n9, C18:2n6, C20:2n6, C22:2n6, C18:3n3, C22:6n3 and C20:5n3. Oleic (C18:1n9), linoleic (C18:2n6), linolenic (C18:3n3), palmitic (C16:0) and stearic (C18:0) acids were the most predominant FAs in the NL canola oil produced from different methods of extraction, i.e. SE, EE 1 and EE 2 oils (Fig. 5B). The extraction methods exhibited significant differences in the proportion of SFA, MUFA, n6PUFA and n3PUFA. SE oil had higher levels of SFA, MUFA and n3PUFA, but lower levels of n6PUFA compared to EE 1 and EE 2 oil (Fig. 6A). It is important to note that the SE, EE 1 and EE 2 extraction methods, fall within the standard range for canola oil extraction [45], [46] which is in accordance with the conditions used by the Canola Council of Canada.

These results demonstrate that the extraction methods used can significantly modify or modulate the composition of the oil during processing. Although the FA composition extracted varied between the EE 1, EE 2 and SE methods, they all retained the unique fatty acid profile that was present in NL canola cultivated under cool climatic conditions in a boreal ecosystems. The FA composition of the canola oil and meal can be modulated by processing methods to produce higher value end products. Consequently, the extraction yield and efficiency are important aspects of the processing method used, since they can have major influence on product quality and revenue [4], [47]. In this study, two expeller presses were selected. A single (EE 1) and a double press (EE 2) method. The advantage of the double press expeller method is that it is more efficient than the single press method, resulting in 8-11% of the residual oil content remaining in the canola meal compared to 10-15% oil remaining in the meal following the EE 1 approach. The high temperature in the expeller method can affect the oil quality though it is very efficient in extracting the oil from canola seeds. A temperature of 104 °C (EE 1 and EE 2) or greater is used in the expeller press method which could increase the oxidation level of the oil. Shang, Dong, Strappe, Zhou and Blanchard [7] suggests that other constituents in the oil, such as antioxidant and other minor compounds, may provide EE oils with an effective and high thermal oxidation stability during oil extraction and usage (frying process). In this study, we did not observe any overall differences between EE 1 and EE 2 in the composition or content of the oil recovered, indicating the EE 1 method would be adequate in retaining the unique FA composition in NL canola oil.

### Effect of extraction methods on the level of MAcDG in canola oil

The three canola oil extractions produced from different processing stages, i.e., SE, EE 1 and EE 2 were found to be significantly different in the proportion of MAcDG, MCTG and LCTG contents. EE 2 oil had higher levels of MAcDG, MCTG, and LCTG compared to SE and EE 1 oils (Fig. 6B). It appears as if the extraction temperatures or the EE 2 had a significant effect in increasing the extraction of triglycerides from NL canola oil inclusive of MAcDG, MCTG, and LCTG.

**Fig. 7:**
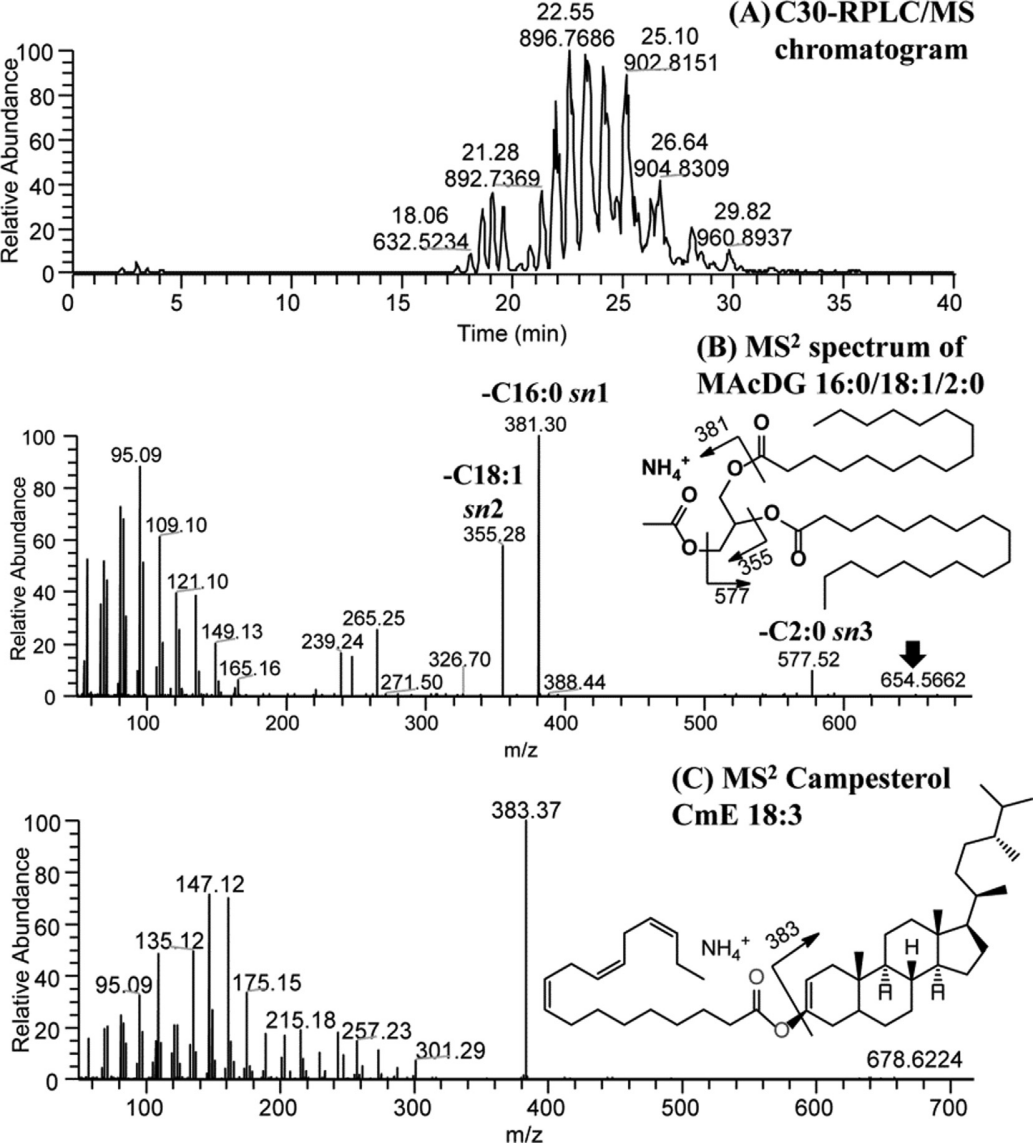
Effects of extraction methods on the level of monoacetyldiacylglycerol (MAcDG) in canola oil. Confirmation of the presence of phytosterols and MAcDG in canola oil using UHPLC-C30RP-HESI-HRAM-MS/MS. Panel (A) shows a full chromatograph of the molecular species in Newfoundland and Labrador (NL) canola oil based on their molecular mass and retention times (B) confirmation of the presence of subclass of a triglyceride in the form of MAcDG (16:0/18:1/2:0) (C) the most predominant sterol (campesterol ester) esterified to a fatty acid (FA) in NL canola oil.

The presence of MAcDG and phytosterols in canola oil was analyzed by UHPLC-MS/MS as demonstrated in Fig. 7A. Despite the different extraction procedures used in the extraction of the oil from the canola seeds (SE, EE 1 and EE 2), their lipid profile remained uniquely intact as measured by the high resolution accurate mass spectrometer employed in this study (Fig. 7). The presence of MAcDG in the lipid profile was detected as ammonium adduct [M + NH_4_]^+^ precursor ions and by the characteristic neutral loss of ammonium acetate (CH_3_COONH_4_ (i.e., −77 Da)). As an example, the MS^2^ of the precursor ion *m*/*z* 654.57 generated three major product ions at *m*/*z* 577.52 (−77 Da), *m*/*z* 355.28 (−299 Da) and 381.30 (−273 Da) in Fig. 7B, representing C2:0, C18:1 and C16:0 FA neutral loss (in the form of ammonium salt), respectively. Fig. 7C shows a typical fragmentation of phytosterol with precursor ion *m*/*z* 678.62, in which *m*/*z* 383.37 represents the campesterol moiety, and a neutral loss of 295 Da corresponding to C18:3 FA. Thus, the structure of the lipid compound (phytosterol) was shown as CmE 18:3 in Fig. 7C.

**Fig. 8:**
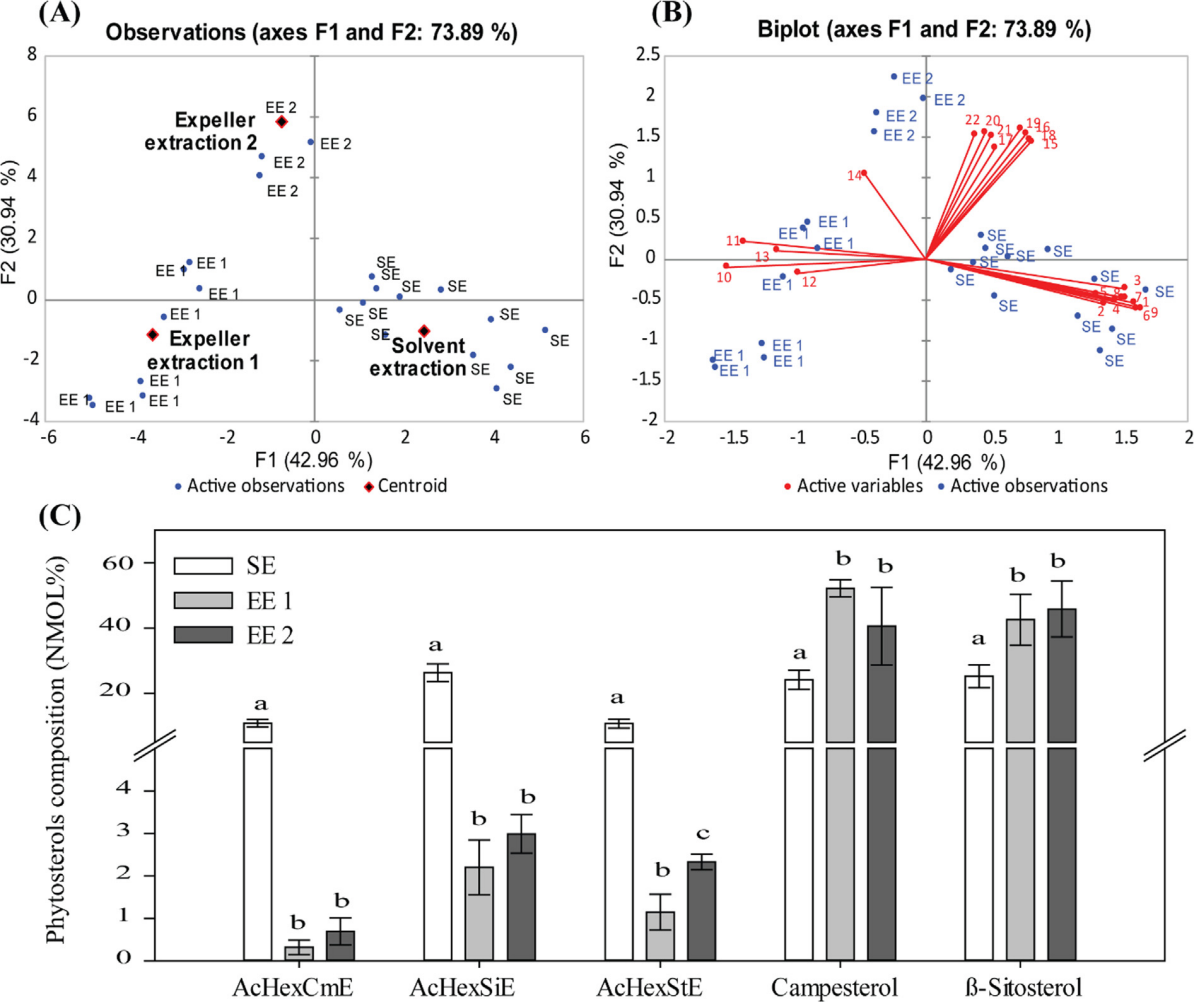
Effects of extraction methods on phytosterols in Newfoundland and Labrador (NL) canola oil. Principal component analysis (PCA) showing (A-B) clustering of the different extraction methods with the monoacetyldiacylglycerole (MAcDG) and phytosterols present in canola oil depending on the extraction methods used. Solvent extraction is indicated as SE, single expeller extraction as EE 1 and double expeller extraction as EE 2. The lipid compositions are represented as the following numbers in the biplot: **1**) AcHexCmE (16:0), **2)** AcHexCmE (18:1), **3)** AcHexCmE (18:2), **4)** AcHexCmE (18:3), **5)** AcHexSiE (0:0), **6)** AcHexSiE (16:0), **7**) AcHexSiE (18:1), **8)** AcHexStE (18:1), **9)** AcHexStE (18:2), **10)** CmE (18:2), **11)** CmE (18:3), **12)** CmE (30:0), **13)** SiE (18:2), **14)** SiE (18:3), **15)** TG (18:1/16:0/2:0), **16)** TG (18:2/16:0/2:0), **17)** TG (18:3/16:0/2:0), **18)** TG (18:1/18:1/2:0), **19)** TG (18:2/18:1/2:0), **20)** TG (18:2/18:2/2:0), **21)** TG (18:3/18:2/2:0), and **22)** TG (18:3/18:3/2:0). **(C)** Effect of oil extraction methods on the phytosterol content in NL canola oil at 104 °C. Results were expressed as means ± standard error. Different lowercase letters above the bar indicate a significant difference (α = 0.05).

PCA was conducted based on the canola oil lipid content of MAcDG and phytosterols, and the results showed the clustering of different extraction methods used in three quadrants of the biplot with the associated lipids extracted (Fig. 8A,B). This segregation accounted for 73.89% of the total variability in the data set. Most of the complex form of phytosterols, e.g., AcHexCmE, AcHexSiE and AcHexStE (compounds **1-9**) were clustered in the same quadrant with canola oil extracted with the SE method, which is consistent with the higher levels recovered using this method of extraction (Fig. 8C). The simple form of phytosterols, e.g., CmE and SiE (compounds **10-14**), however, were more correlated with the EE 1 and EE 2. Interestingly, the levels of MAcDGs (compounds **15-22**) in canola oil were highly associated with EE 2 extraction (Fig. 8B).

The EE 1 is the primary approach used commercially when extracting oils from canola seeds, because it tends to be very efficient [45], [47]. However, the results obtained from this work demonstrates that EE 2 though arduous, extracted more MAcDG, as well as total triglycerides (MAcDG, MCTG, LCTG) from canola (Fig. 6**,8**). Adopting this processing approach for the commercial production of canola oil from NL could be useful in modulating the final oil quality to produce high value NL canola oils. The high value oil could be sold to specialty food or functional foods markets at higher prices thereby increasing the profitability of the farm operation. It also demonstrates the effectiveness of this method in retaining the unique NL canola lipid profile in the oil generated for consumption and commercial use.

### Effect of extraction methods on phytosterols in NL canola oil

SE was more effective in extracting complex phytosterols and this resulted in these phytosterols clustering in the same quadrants of the PCA plots with SE (Fig. 8A,B). Conversely, the simple phytosterols (e.g., CmE and SiE) clustered with the EE 1 and EE 2 methods. This demonstrates the temperature (104 °C) used in expeller processing was effective in the extraction of CmE and SiE from NL canola (Fig. 8C). Furthermore, these findings suggest acylated phytosterols (AcHexCmE, AcHexStE and AcHexSiE) may be more susceptible to degradation at extraction temperatures of 104 °C or greater generated during the expeller press processing of canola oil-seeds. It is of interest to follow the changes in phytosterol content and composition during processing and heating, as this may lend further insights as to how different types of phytosterol composition affect their stability and possible contribution to NL canola oil stability. Studies indicate that the stability of phytosterols is affected by their chemical structure, such as unsaturation in the ring and side chain, temperature, time and composition of the matrix [11], [39]. The output generated from this work, suggest that the processing methods could be used to selectively modulate the types and concentration of phytosterols in NL canola oil. In fact, this could be used to make designer oils with unique phytosterol compositions as long as the phytosterols exist in the canola seeds at the beginning of processing. These findings are very exciting considering the unique lipid profile observed in NL canola, and may present a lot of opportunities to further develop novel NL canola oils and/or NL canola oil-based products with superior functional lipid contents for the commercial food market.

## Conclusion

This study revealed that canola cultivated under boreal climatic conditions had a significantly altered FA composition characterized by a doubling in the level of omega-3 FAs (20%) in NL canola, compared to the standard canola oil globally (9%). Furthermore, we report for the first time the presence of MAcDG in canola. CmE and SiE were the predominant phytosterols found in canola cultivated under low temperatures (11.6 °C) in a boreal climate. Interestingly, SE, EE 1 and EE 2 (104 °C) of canola oil retained the omega-3 content (17-20%), one of the most important dietary FAs. EE 2 was observed to be most effective in retaining the MAcDG content in canola oil. This work demonstrate canola produced under boreal climatic conditions in NL have a unique lipid profile that could be used to develop high value niche canola oil based food products with enhanced omega-3, MAcDG and phytosterols following EE 1 and EE 2. The developed NL canola oil-based food products could have applications in improving human health and nutrition, considering the known roles of omega-3 FAs, phytosterols and MAcDG in reducing the risk factors for common life style related diseases. We hope this work will stimulate future studies in the scientific community to better understand how boreal climate may influence important phytochemicals such as canolol, phenolics, vitamins, tocopherols, protein, lipids, and antioxidants in canola oil; and how the contents of these phytochemicals in boreal climate produced canola may improve human health and nutritional outcomes.

## Compliance with Ethics Requirements

*This article does not contain any studies with human or animal subjects.*

## Declaration of Competing Interest

*The authors declare no conflict of interest.*
