# Supplementary material for: Canola produced under boreal climatic conditions in Newfoundland and Labrador have a unique lipid composition and expeller press extraction retained the composition for commercial use
Source: J Adv Res. 2020 May 18;24:423–34. doi: 10.1016/j.jare.2020.05.002 (PMC7281306; doi:10.1016/j.jare.2020.05.002)
Supplement: Supplementary data 1 [file mmc1.docx]

**SUPPLEMENTARY TABLE**

**Supplementary Table 1** Canola cultivated under boreal climatic conditions at Pasadena, Newfoundland.

| Year | Field size(ha) | Yield (t) | Average (t/ha) | Average NL Temperature (ºC) |
| --- | --- | --- | --- | --- |
| 2016 | 11.3 | 21.3 | 1.88 | 13.88 |
| 2017 | 2.8 | 6 | 2.14 | 11.78 |
| 2018 | 14.2 | 30.9 | 2.18 | 12.18 |

Canola yield was measured in tons per ha (t/ha) for the duration of the study. The typical temperature at the study area was between 7.5 - 17.2 ºC.
